# Evaluation of Bronze Electrode in Electrical Discharge Coating Process for Copper Coating

**DOI:** 10.3390/mi14010136

**Published:** 2023-01-04

**Authors:** JagadeeswaraRao Maddu, Buschaiah Karrolla, Riyaaz Uddien Shaik, Hassan Elahi, Krishnaiah Arkanti

**Affiliations:** 1 Department of Mechanical Engineering, University College of Engineering, Osmania University, Hyderabad 500007, India; 2 Department of Astronautics Electrical and Energy Engineering, University of Rome “La Sapienza”, Via Eudossiana 18, 00184 Rome, Italy; 3 Department of Civil and Environmental Engineering, University of California, Los Angeles, CA 90095, USA; 4 Department of Mechanical and Aerospace Engineering, University of Rome “La Sapienza”, Via Eudossiana 18, 00184 Rome, Italy

**Keywords:** electrical discharge coating, microhardness, material deposition rate, TOPSIS

## Abstract

One of the widely used non-traditional machines for machining of hard materials into complex shapes and different sizes is the electrical discharge machine (EDM). Recently, the EDM has been used for deposition by controlling the input parameters (current and duty cycle). This work was carried out to evaluate the readily available bronze (88% Cu + 12% Sn) electrode for deposition of copper material on titanium alloy. Experiments were conducted according to Taguchi experimental design considering the input parameters of current, Ton, Toff and preheating temperature of substrates. Titanium alloy was further hardened by preheating at temperatures of 100 °C, 300 °C and 500 °C and quenching in brine, castor oil and vegetable oil in order to avoid workpiece erosion. After this treatment, hardness, grain area, grain diameter and number of grains were characterized to compare with pretreated substrates. Then, the treated substrates were taken for copper deposition with the EDM. Output parameters such as material deposition rate (MDR), electrode wear rate (EWR), coating thickness (CT), elemental composition and surface crack density (SCD) were found. Material characterization was carried out using a scanning electron microscope (SEM) with energy dispersive X-ray spectroscopy (EDX) and optical microscopy. Output parameters were optimized with technique for order of preference by similarity to ideal solution (TOPSIS) to find optimum parameters. A sixth experiment with parameter values of Ton of 440 µs, Toff of 200 µs, preheating temperature of 300 °C and quenching medium of castor oil was optimum with MDR of 0.00506 g/m, EWR of 0.00462 g/m, CT of 40.2 µm and SCD 19.4 × 10^7^ µm^2^.

## 1. Introduction

The EDM is a non-traditional machine that supports the fabrication of complex and intrinsic shapes with an excellent surface finish in materials [1]. This is a well-established technique in the fields of biomedical, automotive, chemical, aerospace, tool and die industries [2]. Usually, the EDM removes material by repeated sparks between the workpiece and tool electrode immersed in dielectric medium [3]. The thermal energy between electrode and workpiece creates high temperature plasma, which erodes, melts and evaporates the workpiece material [4]. Meanwhile, the EDC process requires a low current and high duty cycle which reverses the process of the EDM [5]. In EDC, electrode material is deposited on the workpiece with a difference in parameters [6]. Even in EDC, high frequency electrical discharges or sparks cause the workpiece material to melt and vaporize. Extreme temperatures in the range of 8000–12,000 °C lead to erosion and vaporization of workpiece and electrode [7]. Then, material transfer occurs from the electrode to the workpiece under the suitable process conditions and parameter setup. On the surface of the workpiece, a recast layer of redeposited melt materials from the electrode is deposited on the workpiece which is immersed in dielectric medium [8,9]. The deposited material solidifies and forms a coating in dielectric medium. This process modifies the workpiece surface by generating new compositions which can be further processed by quenching and hardening processes [10].

In this work, superalloy Ti6Al4V is exploited as a workpiece due to its essential characteristics, viz., fracture toughness, biocompatibility, improved ductility, wear resistance, yield strength and corrosion resistance [11]. This alloy has proven its applicability in various fields such as medical implants, marine appliances, airframes, automotive industry, etc. Among them, usage of this alloy in some applications, such as medical implants, wastewater treatment plants, etc., requires antibacterial coating [12]. Copper material has proven antibacterial activity since it helps to increase human immunity [13]. So, in this work, copper material was proposed as coating material.

In our previous research, attempts were made to coat copper on titanium alloy using copper electrodes. Firstly, an attempt was made using copper electrodes and it was sparsely coated on the workpiece [14]. Instead, workpiece material was removed and microhole formation was observed. Secondly, brass, which is an alloy of copper (67%) and zinc (33%), was selected to coat copper and a regular, crack free and stable coating of thickness of 22 µm was obtained. In this work, one more attempt is made with a bronze electrode which is also an alloy of copper containing from 0.5 to 11% tin and 0.01 to 0.35% phosphorus.

Bronzes or tin bronzes are alloys containing copper, tin and phosphorus. The addition of tin increases the corrosion resistance and strength of the alloy whereas phosphorus increases the wear resistance and stiffness of the alloy [6]. Phosphor bronzes have high fatigue resistance, solderability, excellent formability and high corrosion resistance. Phosphorus bronze has established applicability in sleeve bearings, cam followers, thrust washers and electrical products such as diaphragms, corrosion resistant bellows and spring washers [14]. This material has proven strength, high wear resistance, fatigue resistance with good machinability and corrosion resistance [15].

Researchers around the world are working to stabilize and standardize the procedure of electrical discharge coatings. Some of the examples are as follows: Algodi et al. [16] have examined the hardness variation of TiC-Fe nanostructured coating by varying the input parameters such as current and Ton and concluded in their study that the latter is the most influencing factor. Mussada et al. [17] have investigated the possibility of PM electrodes for EDM-based surface modification. The investigation was performed in a stepwise manner, though it takes more time, and a good surface finish was obtained. Hsu et al. [18] varied input parameters of the EDM, viz., material removal rate (MRR), surface roughness (Ra) and electrode wear rate (EWR), to improve the surface finishing. Here, oxygen plasma etching treatment was performed to decrease the surface roughness [19]. In order to further increase the surface characteristics, physical vapor deposition (PVD) was performed to coat TiN. Algodi et al. [20] investigated antibacterial coating on titanium alloy by mixing silver nanopowder with dielectric medium and compared it without mixing powder. It was concluded that electrode material deposition is comparatively less when dielectric medium is mixed with silver nanopowder. Tyagi et al. [21] conducted a study to coat a mild steel (MS) workpiece surface with WS2 and copper green compact electrodes in different composition mixing ratios. It was observed that WS2 increases coating thickness whereas current and duty factors influence wear and hardness. Murray et al. [22] reported their work varying the input parameters of EDC to coat different materials of copper, zirconium and tungsten carbide on stainless steel. Bui et al. [23] studied the elemental composition of the modified workpiece surface, tool electrode and dielectric fluid with immersed powder particles. Due to the application of titanium material (Ti6Al4V) in various fields, many studies are ongoing around the world. For instance, Wuyi Ming et al. [24] studied microporosity and microtrench machining, Kahlin et al. [25] studied fatigue behavior of materials, Zhen Zheng et al. [26] worked on laser-induced plasma micromachining and Schnell et al. [27] studied surface topography using femtosecond laser-induced periodic surface structures (FLIPSSs) and micrometric ripples (MRs).

In this work, a bronze electrode was selected to coat copper on titanium alloy (Ti6Al4V) to compare with our previous attempts. Prior to coating, workpiece substrates were preheated at different temperatures of 100 °C, 300 °C and 500 °C and quenched in brine, castor oil and vegetable oil in order to avoid workpiece erosion [28]. After this treatment, hardness, grain area, grain diameter and number of grains were characterized to compare with pretreated substrates. EDC input parameters selected to be optimized were current, Ton, Toff and preheating temperature. TOPSIS techniques were used to optimize the input parameters and material characterization was conducted using SEM with EDX. Explanations about the electrode and workpiece material are provided in Section 2. The experimental procedure is described in Section 3 with a process flowchart. Section 4 explains the results obtained from TOPSIS and material characterization. Section 5 concludes the paper with short conclusions on this work.

## 2. Materials and Methods

### 2.1. Workpiece and Electrode Materials

In this work, titanium alloy was selected as a workpiece due to its applications in various fields, mainly as medical implants, and a bronze electrode was selected as electrode material in order to evaluate it for copper coating [29]. Initially, a plate of titanium was obtained from Ramesh Steels Corporation Pvt. Ltd., Mumbai, India and then substrates of 20 mm × 20 mm × 8 mm were made using a wire-cut EDM, whereas bronze electrodes of 100 mm in length and 10 mm in diameter were made by power-hacksaw. EDM 30 was used as dielectric fluid in this experiment.

The chemical composition, density (kg/m^2^), melting point (°C), specific heat capacity (J/g °C) and hardness of the electrode and substrate are shown in Table 1 [11].

The three levels of EDM machining process parameters selected are shown in Table 2. Output parameters such as surface quality, surface topography and homogeneity of the coatings rely on the input process parameters, viz., current, Ton, Toff and temperature, as shown in Table 2 [19]. Taguchi L9 design was followed to prepare the combination of parameters [30,31] as shown in Table 2.

### 2.2. Output Process Parameters

In this work, output process parameters considered for optimization are material deposition rate (MDR) [32], electrode wear rate (EWR) [33,34] and surface crack density (SCD) [25]. MDR can be represented as

(1) $$\mathrm{MDR}=\frac{\mathrm{WBM}\;-\mathrm{WAM}\;}{\mathrm{Time}}\left(\mathrm{gram}/\min\right)$$

where WAM = weight after machining and WBM = weight before machining.

EWR can be represented as

(2) $$\mathrm{EWR}=\frac{\mathrm{EBM}\;-\mathrm{EAM}\;}{\mathrm{Time}}\left(\mathrm{gram}/\min\right)$$

where EAM = weight of electrode after machining and EBM = weight of electrode before machining.

Finally, surface crack density was considered which can represented as follows:

(3) $$\mathrm{SCD}\;=\frac{T_{l}}{A_{i}}\;\left(\mu m/{\mu m}^{2}\right)$$

where Tl is total crack length in μm and Ai is image area in ${\mu m}^{2}$.

Every researcher is interested in this parameter to provide crack free coating since it is the proper measure of cracks. This parameter depends upon the coefficient of thermal expansion of coating and workpiece material.

### 2.3. Methods

#### TOPSIS

The procedure for TOPSIS optimization is as follows:

**Step 1**: The first step is to create a decision matrix. This method consists of alternatives in the rows and attributes in the columns. The matrix format can be expressed as follows [19,20,21,22,23,24,25,26,27,28,29,30,31,32,33,34,35].

(4) $$D=\;\;a1\;.\;m\;\left[X11\;\cdots \;X1n\;\vdots \;\ddots \;\vdots \;Xm1\;\cdots \;Xmn\;\right]$$

Here, *a* (*i* = 1,2,3,…,*m*) = all possible alternatives, *x* (*j* = 1,2,3,…,*n*) = the attributes related to performance of alternatives, *j* = 1,2,3,…,*n* and *x_ij_* represents the performance of *i* with respect to attribute *j*.

**Step 2**: In this step, normalization of the above decision matrix is carried out and we obtain a normalized decision matrix$\;\gamma_{ij}$. The formula for *r_ij_* is given below:

(5) $$\gamma_{ij}=\frac{-x_{ij}}{\sqrt{\sum_{i=1}^{m}x_{ij}^{2}}}$$

**Step 3:** Here, weights are assigned according to the importance and the weighted normalized decision matrix can be calculated by using the formula V $=\;w_{j}\gamma_{ij}$ *w · r*.

(6) $$V=\left[v_{iJ}\right]$$

(7) $$\overset{n}{\underset{j=1}{\sum }}w_{j}=1$$

**Step 4:** Positive and negative ideal solutions are calculatedtion is calculated by using the follow in this step. The solutions can be represented as the positive ideal (best) solution [36].

(8) $$a^{+}=\left\{\left(v_{ij}\;,j\in J\right)\left(v_{ij}\dot{J}\;\in J\right)J\right\}$$

$$\;v=\{v1^{+},v2^{+},v3^{+}\ldots \ldots .vj^{+}\ldots \ldots vn^{+}\}$$

(9) $$a^{-}=\left\{\left(v_{ij}\;,j\in J\right)\left(v_{ij}\dot{J}\;\in J\right)J\right\}$$

$$\;\;v=\left\{v1^{-},v2^{-},v3^{-}\ldots \ldots .vj^{-}\ldots \ldots vn^{-}\right\}$$

Here, $J=\left\{j=1,2,3,\ldots \ldots .n\right\},\;J^{\prime }=\left\{j=1,2,3,\ldots n\right\}$.

*J* and *J’* are associated with the beneficial and non-beneficial attributes.

**Step 5:** Here, the Euclidean distance of each alternative from the positive and negative ideal solution is calculated by using the following equations:

(10) $$D_{i}^{+}=\overset{n}{\underset{i=1}{\sum }}{\left(v_{ij}-v_{i}^{+}\right)}^{2},\;\;i=1,2,3,\ldots ,m$$

(11) $$D_{i}^{-}=\overset{n}{\underset{i=1}{\sum }}{\left(v_{ij}-v_{i}^{-}\right)}^{2},\;\;i=1,2,3,\ldots ,m$$

**Step 6:** Here, relative closeness to the ideal solution for each alternative is calculated by using the equation is given below:

(12) $$C_{i}^{+}=\frac{D_{i}^{-}}{D_{i}^{+}+D_{i}^{-}},\;i=1,2,3,\ldots ,m;0\leq C_{i}^{+}\leq 1$$

**Step 7:** In the final step, ranking according to the preference order is given. The alternative with maximum relative closeness should be the best choice. +*C_i_* is multi-performance characteristic index (MPCI) in TOPSIS.

## 3. Experimental Procedure

**Figure 1:**
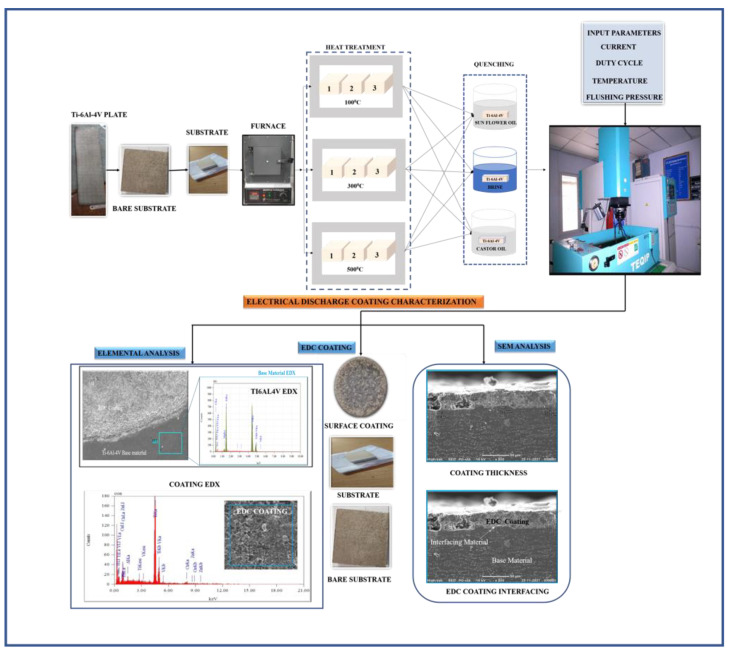
EDC Process Flowchart.

The EDM at the Production Engineering Lab, Osmania University was used for coating. This machine is of CREATER make and numerical control (CNC) is shown in the process flow diagram. Firstly, titanium substrates were ground and polished with emery papers of 50, 100 and 200 micrometers. Then, the substrates were taken for preheat treatment at temperatures of 100 °C, 300 °C and 500 °C and quenched in brine, castor oil and vegetable oil in order to avoid workpiece erosion. Taguchi L9 was followed for heating temperatures as shown in Table 3. Preheat treatment was performed to increase the hardness that prevents workpiece erosion when coating. Before and after the heat treatment, hardness, grain size and grain area of each substrate were measured. Then, the substrates were taken for deposition following the input parameters shown in Table 3. Figure 1 depicts the steps followed in this work for coating copper on titanium alloy. Table 3 shows the MDR and EWR and Table 4 and Table 5 shows the average hardness, average diameter, average grain area, average grain number and grain structure. 

## 4. Results

This section covers the results obtained from the experiments and discussions on analysis of output parameters, surface integrity, surface characterization, surface crack density, coating interface analysis, elemental analysis and optimization by TOPSIS.

### Analysis of Output Parameters

MDR, EWR, CT, elemental analysis and SCD are the output parameters considered in this study [37]. In EDC, output parameters are required to be studied since they depend on the input parameters and finding desired values for input parameters with respect to output parameters is difficult. Conditions to be followed for these parameters are: the higher the better for MDR, the lower the better for EWR and the lower the better for SCD [38]. From the literature, it was observed that lower values of EWR and SCD can be obtained by lower current, pulse-on time, pulse-off time and temperature [39]. According to the design of experiments, it is not possible to select the desired parameter values so there is a requirement of optimization techniques for this problem.

**Figure 2:**
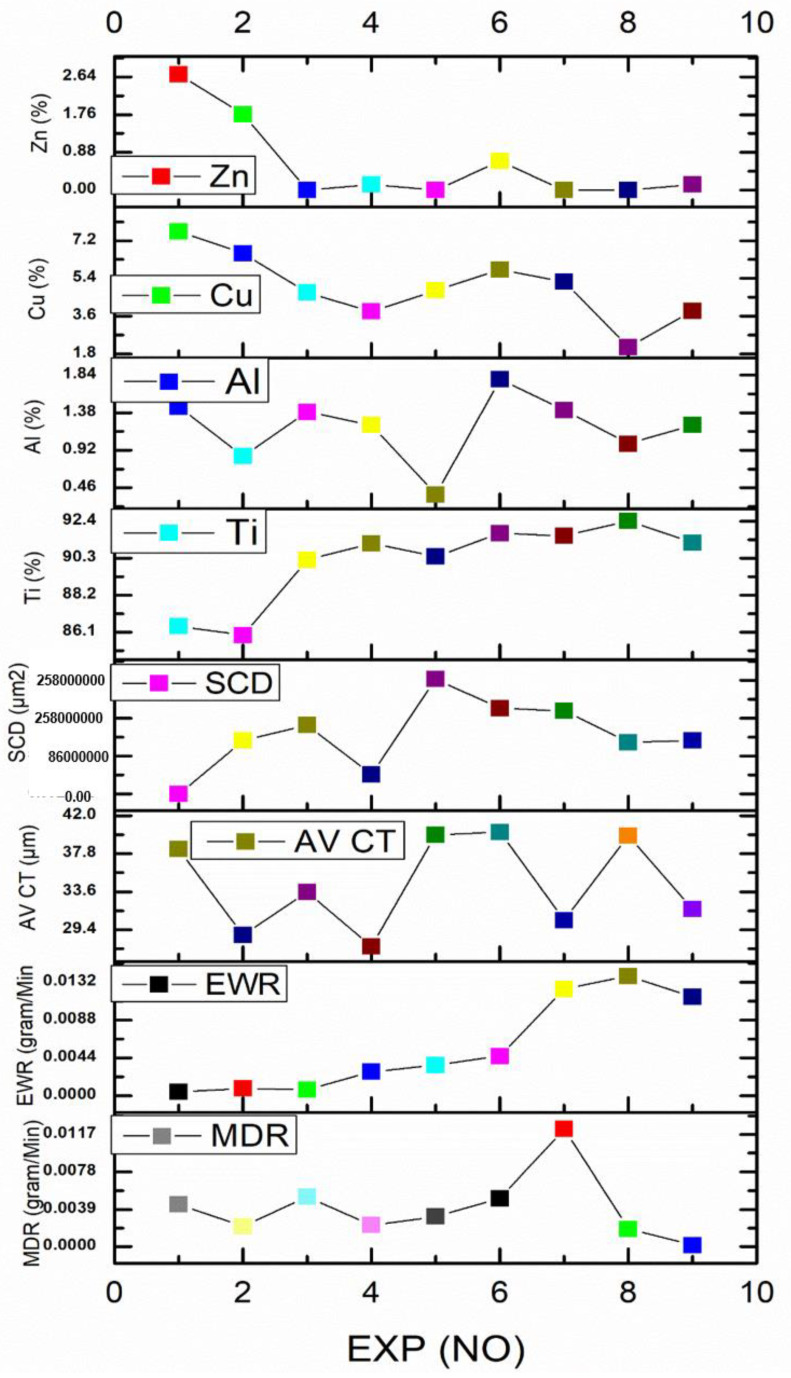
Experimental Graph.

Table 6 shows the MDR, EWR, CT, SCD and elemental analysis obtained for all the experiments in which the maximum MDR and CT are 0.0122775 gram/min and 39.9 µm, respectively, whereas minimum values are 0.00044249 gram/min and 0.0000992775 µm^2^, respectively. Figure 2 shows the graph of output parameters created with the table. Variation in output parameters with respect to variation in input parameters can be observed from Figure 2.

## 5. Discussion

### 5.1. Analysis of Surface Integrity before and after Heat Treatment

**Figure 3:**
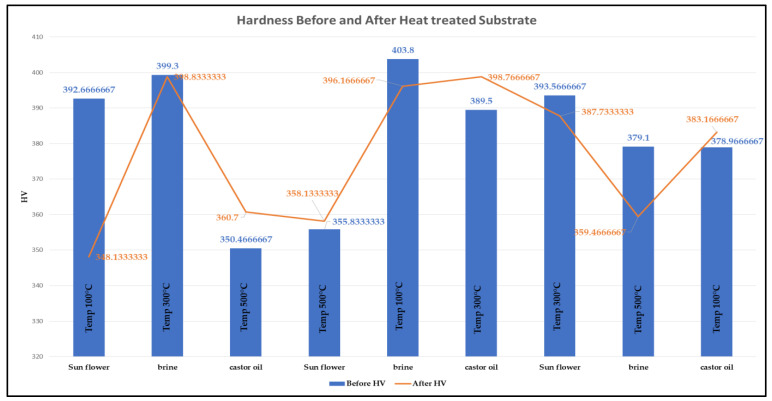
Hardness Before and After heat treatment of substrates.

For the study of surface integrity, all the substrates were measured for hardness individually and showed variation in average hardness ranging from 348 HV to 398 HV taken at six points. For the similar substrates, hardnesses were again measured after heat treatment. From Figure 3, it can be seen that experiments 3, 4, 6 and 9 have shown an increase in hardness after heat treatment at 100 °C, 300 °C, 300 °C and 500 °C, respectively, and quenched in castor oil, vegetable oil, castor oil and castor oil, respectively. An important observation among these was that all substrates quenched in brine solution have shown a decrease in hardness [40]. An increase of around 10 HV after heat treatment at 100 °C with quenching in castor oil was seen, so these parameters were selected.

### 5.2. Surface Morphology

**Figure 4:**
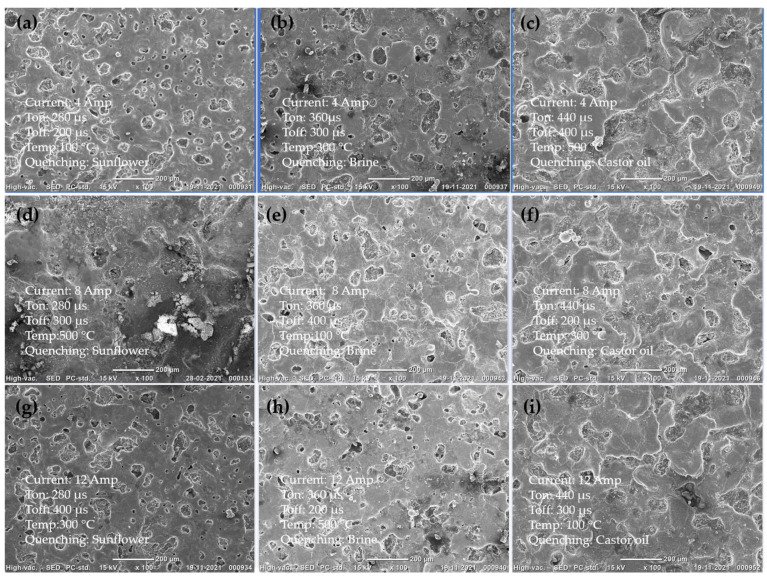
EDC coating Surface observations: (**a**) EX 1, (**b**) EX 2, (**c**) EXP 3, (**d**) EX 4, (**e**) EX 5 and (**f**) EX 6, (**g**) EX 7, (**h**) EX 8 and (**i**) EX 9 (all SEM images are 100X).

Figure 4 shows the SEM images of EDCs developed using different combinations of input parameters with the L9 orthogonal array. While designing the experiments, the process of heating treatment and quenching medium were also considered. It is observed from Figure 4 that all the coatings have a cauliflower structure and uniform coating. Figure 4b,d,g show uneven coating surfaces and machining spatters can be observed. Figure 7c shows uniform coating on the substrate at 100 °C with quenching in castor oil. Input parameters for experiment 3 are current of 4 Amp, Ton of 440 µs and Toff of 400 µs.

### 5.3. Surface Crack Density

**Figure 5:**
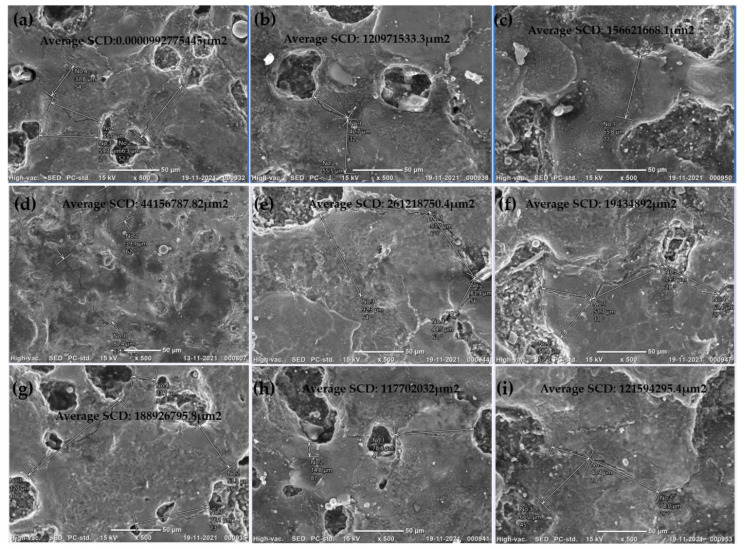
EDC coating Surface with observed microcracks: (**a**) EX 1, (**b**) EX 2, (**c**) EXP 3, (**d**) EX 4, (**e**) EX 5 and (**f**) EX 6, (**g**) EX 7, (**h**) EX 8 and (**i**) EX 9 (all SEM images are 500X).

Figure 5 shows the surfaces of coatings captured using the scanning electron micro-scope. Coatings were thoroughly examined using SEM and if a crack was found, it was zoomed into with a magnification of 500X. Crack length was measured with SEM and SCD was calculated for all the coatings as per Equation (3) [41]. Cracks were observed in almost all the coatings and a minimum crack density of 0.000099277 µm/µm^2^ was ob-tained for Figure 5a.

### 5.4. Coating and Base Material Interfacing Analysis

**Figure 6:**
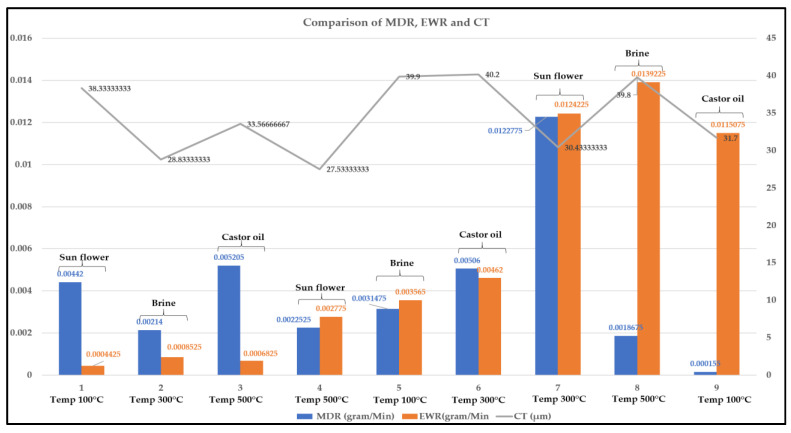
Comparison of MDR, EWR and CT.

**Figure 7:**
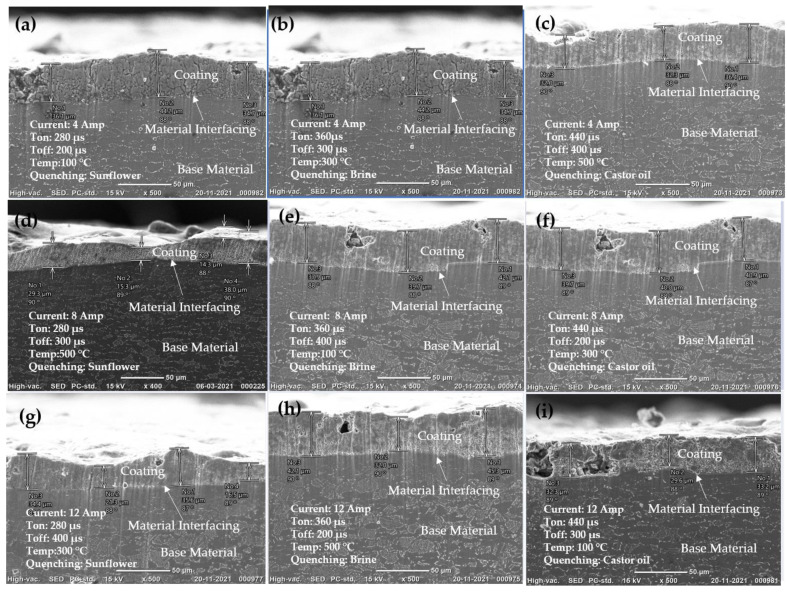
EDC interaction between the base material and coating: (**a**) EX 1, (**b**) EX 2, (**c**) EXP 3, (**d**) EX 4, (**e**) EX 5 and (**f**) EX 6, (**g**) EX 7, (**h**) EX 8 and (**i**) EX 9 (all SEM images are 500X).

Figure 6 depicts the interfacing and bonding of coating on the base material. The investigation of copper coatings obtained on preheated substrates showed major variations in the CT which is also a function of process conditions. SEM images of the cross section of coatings deposited under different conditions are shown in Figure 6. It was observed that with an increase in current, heat is generated and damages the base material, as shown in Figure 6e,g,i. For these experiments, the deposition rate was high due to the current and duty cycle. The highest CT of 40.2 µm can be observed from Figure 6f but it seems to be highly discontinuous with the parameter combination of current of 8 Amp, Ton of 440 µs, Toff of 200 µs, preheated temperature of 300 °C and quenching in castor oil. Figure 7 shows the graph with CTs along with MDR and EWR. From this figure, it can be observed that higher CT does not necessarily mean high MDR and EWR.

### 5.5. Elemental Analysis

**Figure 8:**
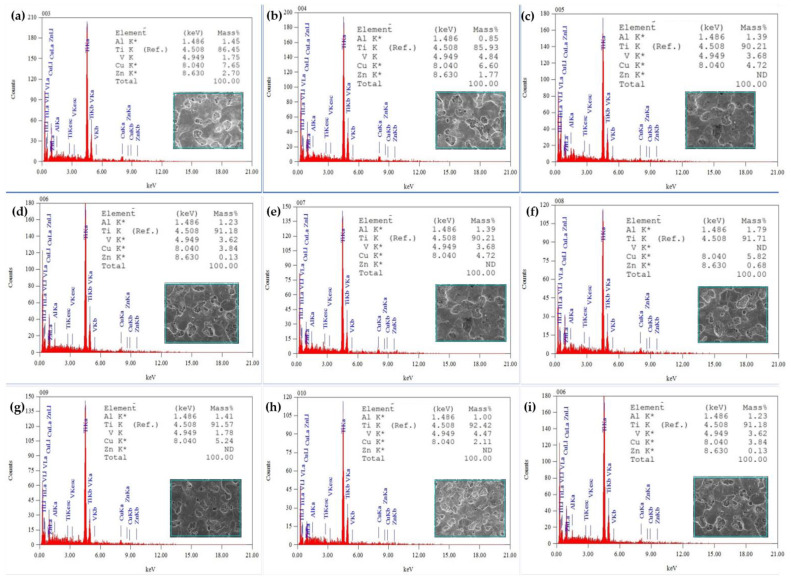
EDC Coating Surface EDX: (**a**) EX 1, (**b**) EX 2, (**c**) EXP 3, (**d**) EX 4, (**e**) EX 5 and (**f**) EX 6, (**g**) EX 7, (**h**) EX 8 and (**i**) EX 9.

SEM was used to inspect the composition of obtained coatings with energy dispersive X-ray spectroscopy (EDX). It was understood from Figure 8 that a higher Ti and Cu percentage was obtained in the coating deposited with the parameters of experiment 8 (Figure 8h), being Ti 92.42%, current 12 Amp, Ton 360 µs, Toff 200 µs and temperature 500 °C and with quenching in brine solution. Meanwhile, the substrate that had the highest copper percentage (7.65%) was coated with the input parameters of current 4 Amp, Ton 280 µs, Toff 200 µs, temperature 100 °C and quenching in sunflower oil.

### 5.6. Optimization by TOPSIS

From the discussion, it can be understood that an optimization technique is required to select the optimum coating among all these coatings. It is difficult to select one manually because each experiment was best with some output parameter. So, the TOPSIS optimization technique was applied to select the optimum coating [42]. TOPSIS is an optimization technique involving seven steps [4]. The formula used to calculate at each step was described in the Section 2. The first step is to form a matrix using the output parameters that support simplifying and processing easily and efficiently, as shown Table 7. Then, further steps were followed as per Section 4.

The last step is to calculate the relative closeness using the formulae shown in Equation (12). The values are tabulated in Table 8, and it can be observed that experiment 6 has higher closeness and takes the rank of 1. This represents the coating obtained from experiment 6 which is the optimum coating as per the conditions of the higher the better MDR and the lower the better EWR and SCD.

## 6. Conclusions

In this work, a bronze electrode was selected to coat copper on titanium alloy (Ti6Al4V) to compare with our previous attempts. Prior to coating, workpiece substrates were preheated at different temperatures of 100 °C, 300 °C and 500 °C and quenched in brine, castor oil and vegetable oil in order to avoid workpiece erosion. After this treatment, hardness, grain area, grain diameter and number of grains were characterized to compare with pretreated substrates. EDC input parameters selected to be optimized were current, Ton, Toff and preheating temperature. The TOPSIS technique was used to optimize the input parameters and material characterization was conducted using SEM with EDX. Some of the conclusions from this study are as follows:

Experiments were carried out according to the Taguchi L9 design of experiments. A higher increase (10 HV) in hardness was obtained for substrate heat treated at 100 °C and quenched in castor oil. It was observed that MDR increases with a decrease in current and EWR increases with an increase in current and Ton.

Surface morphology of all coatings showed a cauliflower structure.

SEM with EDX confirmed a maximum copper percentage of 7.65% in the coating surface whereas copper coated with brass electrodes in our previous study had up to 70% copper material when experiments were performed with the same experimental conditions. The highest coating thickness of 40.2 µm was obtained for experiment 6 when observed in SEM images of magnification of 500X.

Finally, TOPSIS has ranked experiment number six with the input process parameters of current 8 Amp, Ton 440 µs, Toff 200 µs, temperature 300 °C and quenching medium of castor oil as the optimum. Output response values of the same experiments are MDR 0.00506 gram/min, EWR 0.00462 gram/min, CT 40.2 µm and SCD 194348924.µm^2^, with Ti 91.71%, Cu 5.82%, Zn 0.68% and hardness 398.767 HV.

## Figures and Tables

**Table 1:** Physical and Chemical Composition of Electrode and Substrate.

Properties	Electrode	Substrate
Chemical composition	Cu 88% Sn 10% Zn 2%	C 0.08, Fe 0.25, Al 6, V 4, Ti balance
Density (kg/m^3^)	8770	4.42
Melting point (°C)	1035	1878
Specific heat capacity (J/g °C)	370	553
Hardness	170	300

**Table 2:** EDM Input process parameters.

S. No.	Input Process Parameters	Level
1	Current (Amps)	4 8 12
2	Ton (µs)	280 360 440
3	Toff (µs)	200 300 400
4	Temperature (°C)	100 300 500

**Table 3:** Substrate Heating and Quenching and Experimental Data.

Exp. No.	Current (Amp)	Ton (µs)	Toff (µs)	Temp (°C)	Quenching Medium	MDR (Gram/Min)	EWR (Gram/Min)
1	4	280	200	100	Sunflower	0.00442	0.000442
2	4	360	300	300	Brine	0.00214	0.000852
3	4	440	400	500	Castor oil	0.005205	0.000682
4	8	280	300	500	Sunflower	0.002253	0.002775
5	8	360	400	100	Brine	0.003148	0.003565
6	8	440	200	300	Castor oil	0.00506	0.00462
7	12	280	400	300	Sunflower	0.012278	0.012423
8	12	360	200	500	Brine	0.001868	0.013923
9	12	440	300	100	Castor oil	0.000155	0.011508

**Table 4:** Before Heating Hardness and Grain size.

Exp. No.	Avg. HV	Avg. Diameter (Micron)	Avg. Grain Area (Micron Sqr)	Avg. Grain No.	50X	Grain Structure
1	392.66	61.95	4865	4	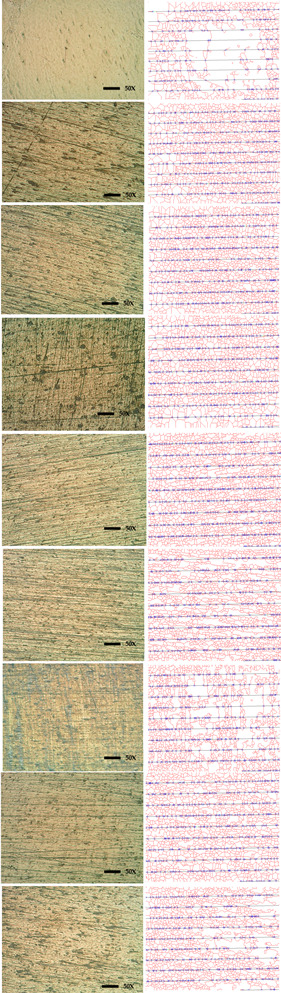	
2	399.3	26.85	861.5	7		
3	350.46	26.85	861.5	7		
4	355.83	30.95	1220	6		
5	408.3	26.85	861.5	7		
6	389.5	30.95	1220	6		
7	393.56	30.95	1220	6		
8	379.1	26.85	861.5	7		
9	378.96	36.8	1725	6		

**Table 5:** After Heating Hardness and Grain size.

Exp. No.	Avg. HV	Avg. Diameter (Micron)	Avg. Grain Area (Micron Sqr)	Avg. Grain No.	50X	Grain Structure
1	348.13	26.85	861.5	7	** 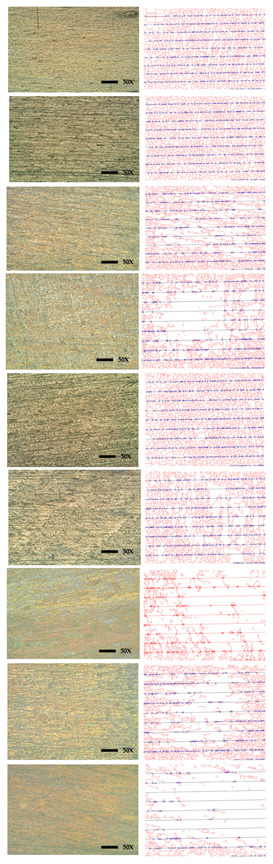 **	
2	398.83	26.85	861.5	7		
3	360.7	30.95	1220	6		
4	358.13	36.8	1725	6		
5	396.166	26.85	861.5	7		
6	398.76	26.85	861.5	7		
7	387.73	52.1	3440	5		
8	359.46	36.8	1725	6		
9	383.16	104.1	13750	3		

**Table 6:** Output response parameters.

Exp. No. Units	MDR (Gram/Min)	EWR (Gram/Min)	CT (µm)	SCD (µm^2^)	Ti %	Al %	Cu %
1	0.00442	0.00044	38.3333	0.0000992775	86.45	1.45	7.65
2	0.00214	0.00085	28.8333	120971533.3	85.93	0.85	6.6
3	0.0052	0.00068	33.5667	156621668.1	90.21	1.39	4.72
4	0.00225	0.00278	27.5333	44156787.82	91.14	1.23	3.82
5	0.00315	0.00357	39.9	261218750.4	90.41	0.38	4.82
6	0.00506	0.00462	40.2	194348924	91.71	1.79	5.82
7	0.01228	0.01242	30.4333	188926795.8	91.57	1.41	5.24
8	0.00187	0.01392	39.8	117702032.4	92.42	1	2.11
9	0.00015	0.01151	31.7	121594295.4	91.18	1.23	3.84

**Table 7:** Decision matrix.

Exp. No.	MDR	EWR	AV CT	SCD	Ti	Cu
1	0.00442	0.000442	38.33333	0.0000993	86.45	7.65
2	0.00214	0.000852	28.83333	121000000	85.93	6.6
3	0.005205	0.000682	33.56667	157000000	90.21	4.72
4	0.002252	0.002775	27.53333	44156788	91.14	3.82
5	0.003147	0.003565	39.9	261000000	90.41	4.82
6	0.00506	0.00462	40.2	194000000	91.71	5.82
7	0.012278	0.012423	30.43333	189000000	91.57	5.24
8	0.001868	0.013923	39.8	118000000	92.42	2.11
9	0.000155	0.011508	31.7	122000000	91.18	3.84

**Table 8:** Relative closeness.

Exp. No.	Relative Closeness	Rank
1	0.370044	3
2	0.370065	9
3	0.370053	5
4	0.370064	8
5	0.370041	2
6	0.370034	1
7	0.370054	6
8	0.370046	4
9	0.370057	7

## Data Availability

Not applicable.
